# p53-independent structure-activity relationships of 3-ring mesogenic compounds’ activity as cytotoxic effects against human non-small cell lung cancer lines

**DOI:** 10.1186/s12885-016-2585-6

**Published:** 2016-07-25

**Authors:** Saori Fukushi, Hironori Yoshino, Atsushi Yoshizawa, Ikuo Kashiwakura

**Affiliations:** 1 Department of Frontier Materials Chemistry, Graduate School of Science and Technology, Hirosaki University, 3 Bunkyo-cho, Hirosaki, Aomori 036-8561 Japan; 2 Department of Radiation Science, Hirosaki University Graduate School of Health Sciences, 66-1 Hon-cho, Hirosaki, Aomori 036-8564 Japan

**Keywords:** Non-small cell lung cancer, Structure–activity relationship, p53, G2/M arrest, G1 arrest, Cell death, Caspase, DNA damage-signaling pathway, Alkyl chain length

## Abstract

**Background:**

We recently demonstrated the cytotoxicity of liquid crystal precursors (hereafter referred to as “mesogenic compounds”) in the human non-small cell lung cancer (NSCLC) cell line A549 which carry wild-type p53. p53 mutations are observed in 50 % of NSCLC and contribute to their resistance to chemotherapy. To develop more effective and cancer-specific agents, in this study, we investigated the structure–activity relationships of mesogenic compounds with cytotoxic effects against multiple NSCLC cells.

**Methods:**

The pharmacological effects of mesogenic compounds were examined in human NSCLC cells (A549, LU99, EBC-1, and H1299) and normal WI-38 human fibroblast. Analyses of the cell cycle, cell-death induction, and capsases expression were performed.

**Results:**

The 3-ring compounds possessing terminal alkyl and hydroxyl groups (compounds C1–C5) showed cytotoxicity in NSCLC cells regardless of the p53 status. The compounds C1 and C3, which possess a pyrimidine at the center of the core, induced G2/M arrest, while the compounds without a pyrimidine (C2, C4, and C5) caused G1 arrest; all compounds produced caspase-mediated cell death. These events occurred in a p53-independent manner. Furthermore, it was suggested that compounds induced cell death through p53-independent DNA damage-signaling pathway. Compounds C2, C4, and C5 did not show strong cytotoxicity in WI-38 cells, whereas C1 and C3 did. However, the cytotoxicity of compound C1 against WI-38 cells was improved by modulating the terminal alkyl chain lengths of the compound.

**Conclusions:**

We showed the p53-indepdent structure–activity relationships of mesogenic compounds related to the cytotoxic effects. These structure–activity relationships will be helpful in the development of more effective and cancer-specific agents.

**Electronic supplementary material:**

The online version of this article (doi:10.1186/s12885-016-2585-6) contains supplementary material, which is available to authorized users.

## Background

Lung cancer is the leading cause of cancer-related death over the world among both men and women. Non-small cell lung cancer (NSCLC) accounts for 85 % of all cases of lung cancer, and the overall 5-year survival rate of patients with NSCLC remains lower than 15 % [1]. To improve the survival of patients with NSCLC, anticancer agents such as molecular-targeted drugs [2–4] are under development. However, few drug therapies lead to complete recovery in patients with NSCLC. Therefore, development of more effective anticancer drugs is essential for the treatment of NSCLC.

p53 is a tumor suppressor gene that plays critical roles in cellular responses, such as cell cycle arrest and apoptosis, after exposure to various stresses including DNA damage [5]. In response to DNA damage such as ionizing radiation, ataxia–telangiectasia mutated/ataxia–telangiectasia and Rad-3-related (ATM/ATR), which is a DNA damage sensor, stabilizes and activates p53; activated p53 then transcriptionally regulates apoptosis-related genes as well as cell cycle arrest-related genes [6]. In addition to transcriptional activity, p53 can activate the intrinsic mitochondrial-mediated pathway of apoptosis in a transcriptional-independent manner by interacting with B-cell lymphoma file family members [7]. The importance of p53 in cancer treatment has been shown in many studies [8–11]. For example, the loss of p53 function in lung cancers results in resistance to not only radiation but also molecularly targeted drugs such as epidermal growth factor receptor inhibitors [10, 11]. This is at least in part due to the impairment of p53-mediated apoptosis induction [12, 13]. Since p53 mutations are observed in 50 % of NSCLC [14] and contribute to their resistance to chemotherapy [15], drugs exerting anticancer effects independent of p53 are required for NSCLC treatment.

Liquid crystals (LCs) are compounds that exist in a state of matter between liquid and crystalline phases and can be characterized by the loss of positional order while maintaining orientational order [16]. Lyotropic LCs can be found in the LC phase depending on both the temperature and the concentration of LC molecules in a solvent; these compounds are observed in biological structures such as cell membranes, which are comprised of a lamellar bilayer of mesophases of phospholipids, glycolipids, and cholesterol. Some studies have focused on the structural affinities of cell membranes for LCs and have assessed the application of LCs as drug-delivery systems [17, 18]. In previous studies, we investigated the cytotoxicity of LC compounds and their precursors (mesogenic compounds) [19–23] and showed that some amphiphilic LC compounds, such as cyanobiphenyl derivatives with terminal hydroxyl moieties, moderately suppressed cell growth in the NSCLC cell line A549 [20]. Furthermore, an amphiphilic LC precursor with three aromatic rings dramatically suppressed cell growth and induced apoptosis in A549 cells, but it also showed low cyototoxicity in normal WI-38 fibroblast cells [22]. To explore and further develop the potential application of LC precursors as anticancer drugs, we investigated the structure–activity relationships of various LC precursors and their analogs and examined their cytotoxic effects in multiple NSCLC cell lines, both with and without p53. Here we demonstrated that not only the 3-ring structure with terminal alkyl and hydroxyl groups but also the alkyl chain lengths are structurally important for the cytotoxic effects of mesogenic compounds against human NSCLC cells. Furthermore, we showed the p53-indepdent structure–activity relationships of mesogenic compounds related to the cytotoxic effects.

## Methods

### Compounds

**Fig. 1:**
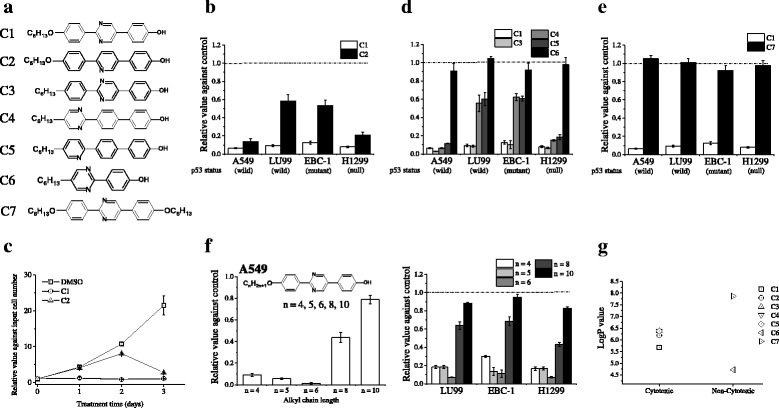
Effects of test compounds on the growth of non-small cell lung cancer (NSCLC) cells. **a** The structures of the test compounds are shown. **b**, **d**–**e** NSCLC cells cultured in the presence of compounds C1–C7 at 10 μM for 3 days were harvested, and viable cells were counted using trypan blue exclusion assays. Dotted lines indicate the dimethyl sulfoxide control. Data are presented as the mean ± SE of 3 independent experiments. **c** A549 cells cultured in the presence of compounds C1–C2 at 10 μM for 1–3 days were harvested, and viable cells were counted using trypan blue exclusion assays. Results are shown as relative value against input cell number. Data are presented as the mean ± SE of 3 independent experiments. **f** Structural formulae of C1 and its derivatives with differing alkyl chain lengths and cytotoxic effects against A549 cells [*left panel*] and the other NSCLC cells [*light panel*]; data are presented as mean ± SE of 3 independent experiments. **g** The logP values of test compounds are shown

Test compounds (Fig. 1a) were dissolved in dimethyl sulfoxide (DMSO; Sigma–Aldrich, St Louis, MO, USA) at 10 mM concentration. 2-(4-Hexyloxyphenyl)-5-(4-hydroxyphenyl)pyrimidine (C1) and its homologous series (as shown in Fig. 1f), 2-(4-hexylphenyl)-5-(4-hydroxyphenyl)pyrimidine (C3), 2-(4-hydroxyphenyl)-5-hexylpyrimidine (C6), and 2,5-bis(4-hexyloxyphenyl)pyrimidine (C7) were purchased from Midori Kagaku Co., Ltd. (Tokyo, Japan). 2-[4-(Hexyloxy)phenyl]-5-(4-hydroxyphenyl)pyridine (C2), 2-[4-(4-hydroxyphenyl)phenyl]-5-hexylpyrimidine (C4), and 2-[4-(4-hydroxyphenyl)phenyl]-5-hexylpyridine (C5) were provided from Japan Energy Corporation (Tokyo, Japan).

The octanol-water coefficient (logP) values were calculated using ChemDraw software (Perkin Elmer Informatics).

### Reagents

Propidium iodide (PI) and caffeine were purchased from Sigma–Aldrich (St. Louis, MO, USA). Z-Val-Ala-Asp (OMe)-CH_2_F (Z-VAD-fmk) was purchased from Peptide Institute, Inc. (Osaka, Japan). The fluorescein isothiocyanate (FITC)-labeled monoclonal antibody anti-human cluster of differentiation 95 (CD95-FITC) was purchased from BioLegend (San Diego, CA, USA). FITC-conjugated anti-mouse IgG_1_ antibody was purchased from Beckman–Coulter (Fullerton, CA, USA). Kp7-6 was purchased from EMD Millipore (Darmstadt, Germany). β-actin antibody (#4967), p53 antibody (#9282), cyclin B1 antibody (#4135), phospho-cdc2 (Tyr15) antibody (#9111), phospho-Histone H3 (Ser10) antibody (#3377), caspase-3 antibody (#9662), caspase-8 antibody (#9746), caspase-9 antibody (#9502), phosphor-ATM (Ser1981) (D25E5) antibody (#13050), anti-rabbit IgG horse radish peroxidase (HRP)-linked antibody (#7074), and anti-mouse IgG HRP-linked antibody (#7076), Alexa Fluor® 488-conjugated goat anti-rabbit IgG (#4412) were purchased from Cell Signaling Technology Japan, K.K. (Tokyo, Japan). Goat anti-actin polyclonal antibody (sc-1615), rabbit anti-p21 polyclonal antibody (sc-756), and HRP-conjugated donkey anti-goat IgG antibody (sc-2056) were purchased from Santa Cruz Biotechnology, Inc. (Santa Cruz, CA, USA). For knockdown of p53, Ambion’s Silencer® select pre-designed siRNA (ID: s606) and Silencer® select negative control 1 were purchased from Life Technologies Corporation (Carlsbad, CA, USA).

### Cell culture and treatment

A549 (p53 wild type) human lung cancer cells and WI-38 normal human fibroblasts (embryonic fibroblast, lung-derived cell line) were purchased from the Riken Bio-Resource Center (Tsukuba, Japan). LU99 (p53 wild type) and EBC-1 (p53 mutant) human lung carcinoma cells were purchased from the JCRB Cell Bank (Osaka, Japan). H1299 (p53-null) human lung carcinoma cells were purchased from the American Type Culture Collection (ATCC, Manassas, VA, USA). The A549 cells were maintained in Dulbecco’s modified Eagle’s medium (Sigma–Aldrich) supplemented with 1 % penicillin/streptomycin (GIBCO®; Invitrogen, CA, USA) and 10 % heat-inactivated fetal bovine serum (FBS; Japan Bioserum Co., Ltd., Japan) at 37 °C in a humidified atmosphere containing 5 % CO_2_. WI-38 and EBC-1 cells were cultured in Eagle’s minimum essential medium (Sigma–Aldrich) supplemented with 1 % penicillin/streptomycin and 10 % heat-inactivated FBS at 37 °C in a humidified atmosphere containing 5 % CO_2_. The LU99 and H1299 cells were maintained in RPMI1640 (GIBCO®) supplemented with 1 % penicillin/streptomycin and 10 % heat-inactivated FBS at 37 °C in a humidified atmosphere containing 5 % CO_2_.

NSCLC cell lines (6.0 × 10^4^ cells) or WI-38 cells (6.0 × 10^4^ cells) were seeded onto 35-mm culture dishes (Iwaki, Chiba, Japan) and were cultured overnight to allow adherence to the dish. On the next day, compounds were added to the culture medium (at a final concentration of 10 μM) and cells were cultured for 3 days. For cell cycle analysis, NSCLC cell lines (1.2 × 10^5^ cells) were seeded onto 60-mm culture dishes (Iwaki, Chiba, Japan). Cells were subsequently harvested using 0.1 % trypsin-ethylenediaminetetraacetic acid (Gibco®; Invitrogen), and viable cells were counted using the trypan blue dye exclusion method. Because LU99 cells were loosely adhered to the dish, they were harvested without the use of trypsin-ethylenediaminetetraacetic acid. Cell survival was expressed as the rate of viable cell number against the vehicle (DMSO)-treated cells. No significant cytotoxicty of DMSO treatment was observed in any of the cell lines.

### siRNA transfection

A549 cells were transfected with p53-targeting siRNA (p53 siRNA) or control siRNA using Lipofectamine RNAiMAX (Invitrogen) according to the manufacture’s recommended protocol. The final concentration of siRNA was 5 nM. After 24 h incubation, the transfected cells were harvested and used for subsequent analyses.

### Cell cycle analysis

The cells treated with compounds were harvested and fixed in 70 % ethanol overnight at −20 °C. The fixed cells were washed with Ca^(2+)^- and Mg^(2+)^- free phosphate-buffered saline [PBS(−)] and then treated with RNase A (200 μg/mL) at 37 °C for 30 min to hydrolyze RNA. After treatment, the cells were washed with PBS(−) and stained with PI (30 μg/mL) for 30 min in the dark. Then, the cells were filtered with cell strainer (BD Falcon™, Franklin Lakes, NJ, USA). The cell-cycle distribution was analyzed by flow cytometry (Cytomics FC500, Beckman–Coulter).

### SDS-PAGE and western blotting

SDS-PAGE and western blotting were performed as previously described [24]. The following primary antibodies were used: rabbit anti-p53 antibody (1:4000), rabbit anti-p21 polyclonal antibody (1:4000), rabbit anti-phospho-Histone H3 antibody (1:3000), rabbit anti-phospho-cdc2 antibody (1:3000), mouse anti-cyclin B1 antibody (1:3000), rabbit anti-caspase-3 antibody (1:3000), mouse anti-caspase-8 antibody (1:3000), rabbit anti-caspase-9 antibody (1:3000), rabbit anti-β-actin antibody (1:5000), or goat anti-actin polyclonal antibody (1:5000). Each primary antibody was diluted in Can Get Signal® Immunoreaction Enhancer Solution 1 (TOYOBO, Co., Ltd, Osaka, Japan). The following secondary were used: HRP-linked anti-rabbit IgG antibody (1:10000), HRP-linked anti-mouse IgG antibody (1:10000), or HRP-conjugated donkey anti-goat IgG antibody (1:10000). Each secondary antibody was diluted in Can Get Signal® Immunoreaction Enhancer Solution 2 (TOYOBO). The antigens were visualized by the ECL Prime Western Blotting Detection System (GE Healthcare). Blot stripping was performed using Stripping Solution (Wako Pure Chemical Industries).

### In vitro irradiation

Radiation exposures (150 kVp, 20 mA, 0.5-mm Al and 0.3-mm Cu filters) were performed using an X-ray generator (MBR-1520R-3, Hitachi Medical Corporation, Tokyo, Japan) at a distance of 45 cm from the focus at a dose rate of 1.04 Gy/min.

### Analysis of apoptosis

Cell death was analyzed by annexin V-FITC (BioLegend) and PI staining according to the manufacturer’s instructions. In brief, the cells treated with each compound were harvested, washed, and suspended in annexin V Binding Buffer (BioLegend). The annexin V-FITC (2.5 μg/mL) and PI solution (50 μg/mL) were added to the cell suspension and incubated for 15 min at room temperature in the dark. Then, the apoptotic cells were analyzed by flow cytometry (Cytomics FC500, Beckman–Coulter). In the annexin V/PI quadrant gating, annexin V(−)/PI(−), annexin V(+)/PI(−), and annexin V(+)/PI(+) were used to identify the fraction of viable cells, early apoptotic cells, and late apoptotic/necrotic cells, respectively.

### Detection of active caspase-3

An FITC-conjugated monoclonal active caspase-3 antibody apoptosis kit (BD Biosciences, San Diego, CA, USA) was used to detect active caspase-3 according to the manufacturer’s instructions. In brief, the cells treated with each compound were harvested, washed, and suspended in Cytofix/Cytoperm™ buffer. The cell suspensions were placed on ice for 20 min and washed with Perm/Wash™. Then, the cells were resuspended in Perm/Wash™ buffer containing 10 % FITC-conjugated caspase-3 antibody and incubated for 30 min at room temperature in the dark. The cells were washed and analyzed by flow cytometry.

### Treatment with various inhibitors

Cells were preincubated with each inhibitor, 50 μM Z-VAD-fmk (a pan-caspase inhibitor), 1 mM kp7-6 (Fas/Fas ligand antagonist), or 2 mM caffeine (an inhibitor of ATM/ATR) for 1 h, and then test compounds were added to the respective cultures. After culture in the presence of the compounds for 24–72 h, the cells were harvested and viable cells were counted. Subsequently, analyses of cell cycle, cell death (annexin V-FITC and PI staining), or caspase-3 expression were performed as described above.

### Analysis of cell surface CD95 (Fas) expression

Cells treated with compounds were harvested and then washed in PBS(−). Cells were then stained with FITC-conjugated CD95 antibody for 30 min at 4 °C in the dark. After staining, cells were washed and analyzed using flow cytometry. Before analysis, 25 μg/ml PI was added to cell suspensions to discriminate dead cells from viable cells. After gating PI negative cells, the fluorescence intensity of CD95 staining was analyzed.

### Intracellular phosphorylated-ATM staining

Intracellular phosphorylated-ATM (Ser1981) expression was analyzed using a flow cytometer. Cells treated with compounds were harvested and then washed with PBS(−). The cells were fixed in 4 % formaldehyde (Sigma–Aldrich) for 10 min at 37 °C. After washing with PBS(−), the cells were permeabilized with 90 % methanol overnight at −20 °C. After washing with incubation buffer (PBS containing 0.5 % bovine serum albumin), cells were suspended in incubation buffer containing primary phosphorylated-ATM antibody (1:1600) for 1 h at room temperature. After washing with incubation buffer, cells were stained with Alexa Fluor® 488-conjugated anti-rabbit secondary antibody (1:1000) at room temperature in the dark. As a control, cells were stained with Alexa Fluor® 488-conjugated secondary antibody alone. After 30 min, cells were washed with incubation buffer and were analyzed using flow cytometry.

### Statistical analysis

Data are presented as mean ± SE. Comparisons between control and experimental groups were made using two-sided Mann–Whitney’s U-test or two-sided Student’s t-test depending on the normality of data distributions. Differences were considered significant when *p* < 0.05. Excel 2010 software (Microsoft, USA) with the add-in software Statcel 3 [25] was used to perform these statistical analyses.

## Results

### Cytotoxic effects of compounds

We have previously showed that 2-(4-butoxyphenyl)-5-(4-hydroxyphenyl) pyrimidine (the compound with 4-carbon alkyl chains in Fig. 1f), which is a LC-related compound, dramatically suppresses the growth of A549 cells [22]. Based on this, we developed the mesogenic compounds C1 and C2 (Fig. 1a). Although the structural difference between C1 and C2 is the presence of a pyrimidine at the center of the core, both of these compounds showed cytotoxicity to all the tested NSCLC cell lines (Fig. 1b). Of note, these cytotoxic effects were independent of p53 status. When the dose–response effects of C1 and C2 were examined in A549 cells (Additional file 1: Figure S1), we found that the 50 % inhibitory concentrations (IC_50_) of these compounds were 1.9 ± 0.1 μM for C1 and 2.3 ± 0.2 μM for C2; the cytotoxic effects of these compounds saturated around 10 μM. Therefore, a concentration of 10 μM was used in subsequent experiments. Furthermore, treatment with C1 and C2 at 10 μM finally gave similar cytotoxicity, although the time dependence of these effects differed between C1 and C2 (Fig. 1c). In brief, C1 showed a dramatic cytotoxicity already at 1 day, while the strong cytotoxicity of C2 was observed at 3 day.

To clarify the structure–activity relationship of mesogenic compounds, we first focused on the core structure (Fig. 1d). The presence of an ether linkage between the terminal alkoxyl and the core did not affect cytotoxicity. Compounds C4 and C5, which possess a nitrogen-containing *p*-terphenyl link, retained cytotoxicity, although we found that the cytotoxic effects varied depending on the presence and position of the pyrimidine. However, 2-ring compounds failed to show cytotoxicity. These results suggested that *p*-terphenyl is important for cytotoxic effects.

Next, we investigated structure–activity relationships in terms of the terminal alkoxyl and hydroxyl groups (Fig. 1e). Compounds with alkoxyl groups at both ends did not show cytotoxicity despite possessing a *p*-terphenyl. We then investigated whether alkyl chain length modulates cytotoxicity (Fig. 1f). Compound C1, with 6-carbon alkyl chains, had the highest cytotoxicity. Compounds with <6-carbon alkyl chains (*n* = 4 or 5) also dramatically suppressed cell growth, but cytotoxic effects decreased when the alkyl chain length exceeded 6 carbons (*n* = 8 and 10). These findings show that the presence of *p*-terphenyl with terminal alkoxyl and hydroxyl groups is structurally important for cytotoxicity.

As the lipophilicity of each compound contributes to properties such as solubility and permeability through biological membranes [26], we compared each compound’s logP as an estimate of a compound’s overall lipophilicity. As shown in Fig. 1g, the cytotoxic compounds C1–C5 had similar logP values (5.68–6.38).

### Mesogenic compounds affect cell cycle distribution

**Fig. 2:**
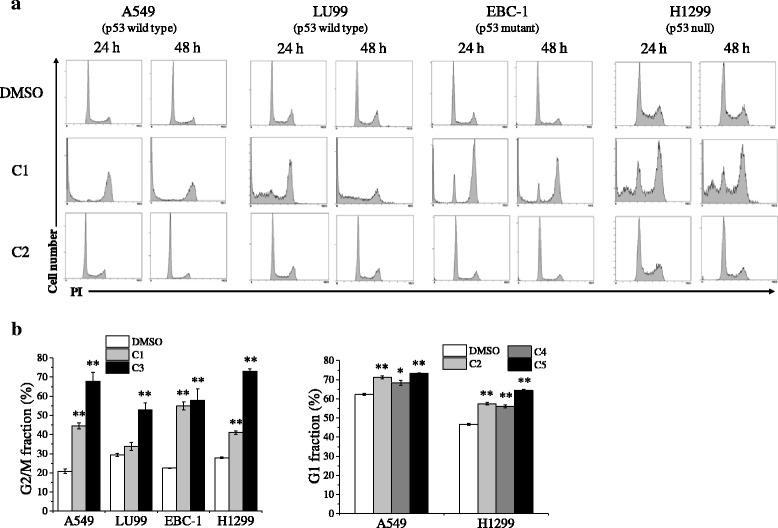
Effects of test compounds on cell cycle progression. **a** Non-small cell lung cancer (NSCLC) cells cultured in the presence of test compounds (C1–C2) at 10 μM for 24–48 h were harvested, and then cell cycle profiles were analyzed. Representative histograms of three different experiments are shown. **b** NSCLC cells cultured in the presence of test compounds (C1–C5) at 10 μM for 24–48 h were harvested, and then cell cycle profiles were analyzed. Fractions of G2/M at 24 h in NSCLC cells (A549, LU99, EBC-1, and H1299 cells) [*left panel*] and G1 at 48 h in A549 and H1299 cells [*light panel*] are shown. Data are presented as mean ± SE of 3 independent experiments. * and ** indicate *p* < 0.05 and *p* < 0.01 compared with dimethyl sulfoxide control, respectively

Since anticancer drugs suppress tumor cell growth in part through modulation of the cell cycle, we next investigated the effects of the cytotoxic compounds C1–C5 on the cell cycle distribution of NSCLC cells. As shown in Fig. 2a, the cell cycle kinetics of NSCLC cells treated with C1 were dramatically different from those treated with C2. Compounds C1 and C3, which have a pyrimidine at the center of the core, induced G2/M arrest (Fig. 2a-b and Additional file 2: Figure S2). In the case of C1, G2/M arrest was induced at 6 and 12 h in LU99 cells (data not shown), although it was not maintained at 24 h in this cell line. In contrast, the compounds without a pyrimidine at the center of the core (compounds C2, C4, and C5) induced G1 cell cycle arrest in A549 and H1299 cells (Fig. 2b). These results indicate that mesogenic compounds carrying *p*-terphenyl with terminal alkoxyl and hydroxyl groups affect cell cycle distribution, and that compounds possessing a pyrimidine at the center of the core induce G2/M arrest regardless of p53 status in NSCLC cells.

**Fig. 3:**
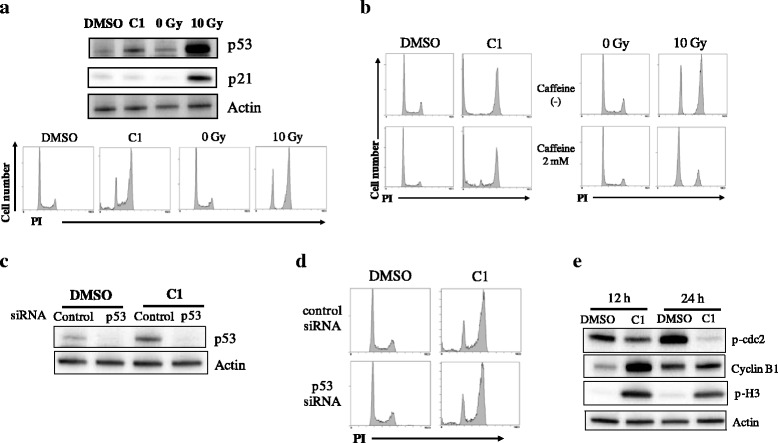
Involvement of p53 in compound C1–induced G2/M arrest. **a** A549 cells were treated with C1 for 12 h and harvested for western blotting of p53 and p21 and cell cycle analyses. As a positive control, A549 cells were irradiated with 10 Gy X-ray and harvested 12 h after irradiation for Western blotting analyses of p53 and p21; actin was used as a loading control. Representative blots of two different experiments are shown [*upper panel*]. Representative histograms of two different experiments are shown [*lower panel*]. **b** A549 cells preincubated with the caffeine were cultured in the presence of 10 μM C1 for 24 h [*left panel*] or cultured for 12 h after 10 Gy-irradiation [*right panel*]. The cells were harvested, and cell cycle analysis was performed. Representative histograms of two different experiments are shown. **c**–**d** A549 cells treated with p53 siRNA were cultured in the presence of C1 at 10 μM for 12 h, and then western blotting of p53 (**c**) and cell cycle profiles (**d**) were analyzed. Representative results of two different experiments are shown. **e** A549 cells were treated with C1 for 12–24 h and harvested for western blotting of phospho-cdc2 (p-cdc2), cyclin B1, and phospho-histone H3 (p-H3). Representative results of two different experiments are shown

Next, to investigate the role of p53 in the G2/M arrest caused by C1 in p53-wild-type A549 cells, we examined the expression levels of p21, which is a transcriptional target of p53 and participates in the G2 checkpoint [27]. Although high levels of p21 expression were observed in irradiated A549 cells, treatment with C1 did not change the expression of p21, despite both treatments leading to G2/M arrest (Fig. 3a). In addition, treatment with caffeine, an ATM/ATR inhibitor [28], hardly affected the C1-induced G2/M arrest, whereas it abrogated the irradiation-induced G2/M arrest (Fig. 3b). These results indicate that ATM/ATR-p53-p21 axis is not involved in the C1-induced G2/M arrest. Furthermore, we analyzed the cell cycle distribution of A549 cells transfected with p53-targeting siRNA. Although knockdown of p53 diminished the C1-induced p53 expression (Fig. 3b), the increase of G2/M fraction after C1 treatment was observed in p53-siRNA trasfected A549 cells (Fig. 3c). These results strongly indicate that C1 induces G2/M arrest in A549 cells in a p53-independent manner.

Cdc2/cyclin B1 kinase plays a key role in regulating the G2/M transition [29–31]. The expression of cyclin B1 began to increase during G2 and peaks in mitosis. On the other hand, dephosphorylation of cdc2 activates the cdc2/cyclin B1 kinase and triggers the cell to enter into mitosis. Since it is reported that a natural plant product curcumin can induce a similar G2/M arrest in human colorectal cancer lines HCT-116 (p53+/+), HCT-116 (p53−/−), and HCT-116 (p21−/−) by regulating cdc2/cyclin B1 kinase activity [32], we investigated the expression of cyclin B1 and phosphorylated-cdc2 (Tyr15) in A549 cells treated with C1. As shown in Fig. 3e, the expression of cyclin B1 was increased after C1 treatment for 12 h, while C1 decreased the phosphorylation of cdc2. These results suggest that cdc2/cyclin B1 kinase is activated after C1 treatment and that C1 treatment induces mitotic arrest rather than G2 arrest. In line with these results, the expression of phosphorylated-histone H3 (Ser10), a marker of mitotic cells, was observed in A549 cells treated with C1 (Fig. 3e). Furthermore, the analysis of nuclear morphology showed the increase of mitotic cells by C1 treatment (Additional file 3: Figure S3). Taken together, these results indicate that C1 induces mitotic arrest.

### Mesogenic compounds cause apoptosis

**Fig. 4:**
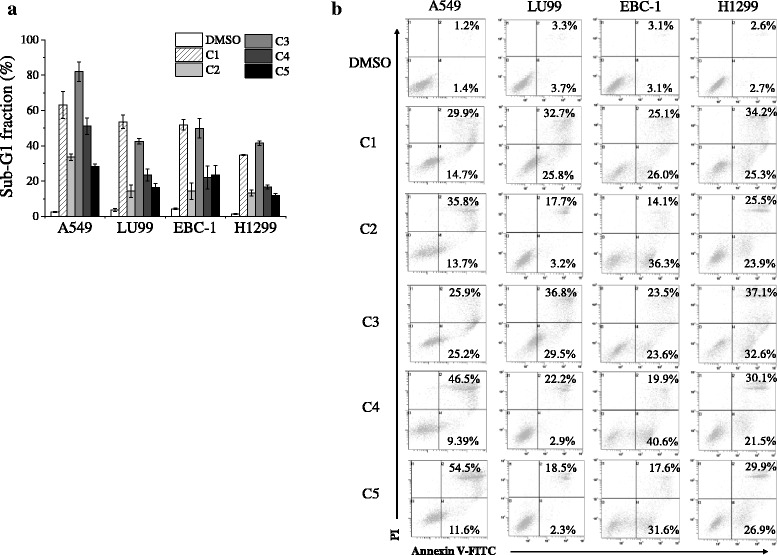
Effects of test compounds on cell death induction. **a** Non-small cell lung cancer (NSCLC) cells cultured in the presence of test compounds (C1–C5) at 10 μM for 72 h were harvested, and then cell cycle profiles were analyzed. Sub-G1 fractions in cells are presented as mean ± SE of 3 independent experiments. **b** NSCLC cells were cultured in the presence of test compounds (C1–C5) at 10 μM for 72 h (A549, LU99, and H1299 cells) or for 96 h (EBC-1 cells). Cells were harvested, and then annexin V/propidium iodide (PI) staining was performed. Representative histograms of three different experiments are shown, and the inset numbers are the percentage of annexin(+)/PI(−) and annexin(+)/PI(+) cells

To further verify the cytotoxic effects of our mesogenic compounds, we estimated apoptosis by sub-G1 fraction or annexin V-FITC and PI staining. As shown in Fig. 4a, treatment with C1–C5 increased the sub-G1 fraction, which is one of the hallmarks of apoptosis, in all NSCLC cell lines. Furthermore, C1–C5 induced annexin V(+)/PI(−) and/or V(+)/PI(+) dead cells (Fig. 4b). These results indicate that mesogenic compounds carrying *p*-terphenyl with terminal alkoxyl and hydroxyl groups induce cell death, including apoptotic death, in NSCLC cells regardless of p53 status.

### Involvement of caspase in mesogenic compound-induced cell death

**Fig. 5:**
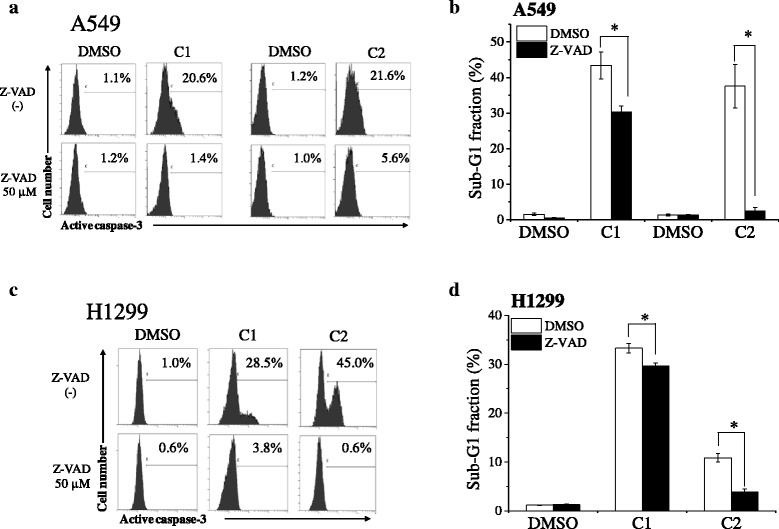
Involvement of caspase in mesogenic compound-induced cell death. **a**–**b** A549 cells were preincubated with Z-VAD-fmk for 1 h and then cultured in the presence of 10 μM C1 for 48 h or C2 for 72 h. Cells were then harvested, and analyses of active caspase-3 expression and sub-G1 fractions were performed. **a** Representative histograms of three different experiments are shown, and inset numbers indicate percentages of active caspase-3-positive cells. Z-VAD indicates Z-VAD-fmk. **b** Sub-G1 fractions are presented as mean ± SE of 4 independent experiments. * indicates *p* < 0.05. **c**–**d** H1299 cells were preincubated with Z-VAD-fmk for 1 h and then cultured in the presence of 10 μM C1 or C2 for 72 h. Cells were then harvested, and analyses of active caspase-3 expression and sub-G1 fractions were performed. **c** Representative histograms of two different experiments are shown, and inset numbers indicate percentages of active caspase-3-positive cells. **d** Sub-G1 fractions are presented as mean ± SE of 3 independent experiments. * indicates *p* < 0.05

Because caspase-3 is a key effector protease responsible for DNA fragmentation during apoptosis, caspase-3 activation was examined. The expression of active caspase-3 was approximately 20 % higher in A549 cells treated with C1 for 48 h or C2 for 72 h than in the control cells, and this expression was reduced in the presence of the pan-caspase inhibitor Z-VAD-fmk (Fig. 5a). In line with the reduction of active caspase-3 expression, treatment with Z-VAD-fmk decreased C1- and C2-induced sub-G1 fractions in A549 cells (Fig. 5b). Furthermore, C1- and C2-induced active caspase-3 expression and sub-G1 fractions in p53-null H1299 cells were decreased by Z-VAD-fmk (Fig. 5c and d). Similar results were observed for C3–C5 treatment (data not shown). These results indicate that mesogenic compounds induce caspase-mediated cell death independent of p53.

### Mesogenic compounds induce Fas-mediated cell death

**Fig. 6:**
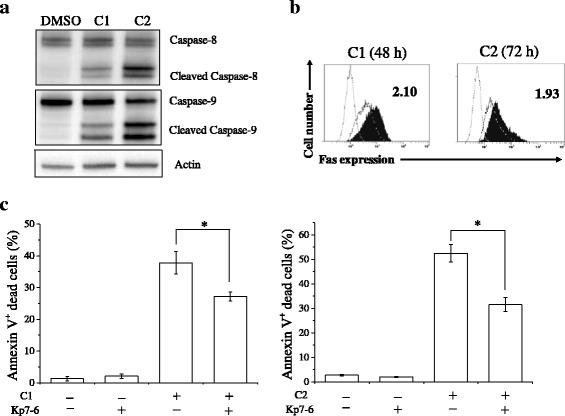
Involvement of Fas in compound-induced cell death of A549 cells. **a** A549 cells were treated with C1 or C2 for 72 h and harvested for western blotting of caspase-8 and caspase-9. Representative results of two different experiments are shown. **b** A549 cells cultured in the presence of 10 μM C1 for 48 h or C2 for 72 h were harvested, and cell surface Fas expression was analyzed. Representative histograms of three different experiments are shown. The dotted line histogram indicates cells stained with isotype control. The broken line and filled gray histograms indicate Fas expression in cells treated with vehicle and compound, respectively. Inset numbers indicate mean fluorescence intensities relative to the vehicle control. **c** A549 cells were preincubated with the Fas/Fas ligand antagonist kp7-6 and were cultured in the presence of 10 μM C1 for 48 h [*left panel*] or C2 for 72 h [*right panel*]. Cells were harvested and annexin V/propidium iodide staining was performed. Percentages of annexin V(+) cells are presented as the mean ± SE of 3 independent experiments; * indicates *p* < 0.05

Since the executioner caspase-3 can be activated by an extrinsic (capase-8) and/or an intrinsic pathway (capsase-9), we analyzed the expression of caspase-8 and -9 in A549 cells treated with C1 and C2. As shown in Fig. 6a, C1 and C2 induced both cleaved caspase-8 and cleaved caspase-9 expression. We next investigated the involvement of Fas in cell death induced by our compounds because Fas induces caspase-mediated cell death [33]. As shown in Fig. 6b, Fas expression in A549 cells treated with C1 for 48 h was approximately 2-fold higher than that in control cells. Furthermore, C1-induced cell death was significantly prevented by treatment with the Fas/Fas ligand antagonist kp7-6 [34] (Fig. 6c), indicating that C1 induces Fas-mediated cell death. Similarly, treatment with C2 increased Fas expression and C2-induced cell death was also decreased by kp7-6 (Fig. 6b and c). These results suggest that 3-ring compounds carrying terminal alkoxyl and hydroxyl groups may possess structurally important features for Fas-mediated cell death that are independent of the central pyrimidine moiety.

### Involvement of DNA damage-signaling pathway in mesogenic compound-induced cell death

**Fig. 7:**
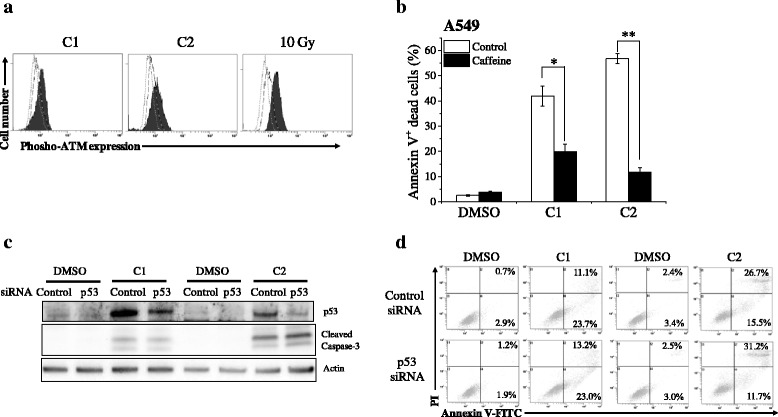
Involvement of DNA damage-signaling pathway and p53 in compounds-induced cell death of A549 cells. **a** A549 cells cultured in the presence of 10 μM C1 for 48 h or C2 for 72 h were harvested, and intracellular phospho-ATM expression was analyzed. Representative histograms of three different experiments are shown. The dotted line histogram indicates cells stained with Alexa Fluor® 488-conjugated secondary antibody alone. The broken line and filled gray histograms indicate phosphor-ATM expression in cells treated with vehicle and compound, respectively. As a positive control, A549 cells were irradiated with 10 Gy X-ray and harvested 30 min after irradiation. **b** A549 cells preincubated with the caffeine (2 mM) were cultured in the presence of 10 μM C1 or C2 for 72 h. The cells were harvested, and annexin V/propidium iodide staining was performed. Percentages of annexin V(+) cells are presented as the mean ± SE of three independent experiments; * and ** indicate *p* < 0.05 and *p* < 0.01. **c**–**d** A549 cells treated with p53-targeting siRNA were cultured in the presence of 10 μM C1 for 48 h or C2 for 72 h. The cells were harvested, and then western blotting (**c**) and cell death analysis (**d**) were performed. Representative results of two different experiments are shown

Since a number of chemotherapeutic drugs cause cytotoxicity through DNA damage [35], we investigate whether mesogenic compounds induce cell death through DNA damage. The serine/threonine protein kinase ATM plays key role in DNA damage response such as DNA repair, cell cycle arrest, and apoptosis [6]. Following DNA damage, ATM activates itself by autophosphorylation at Ser1981 [36]. Once activated, ATM phosphorylates numerous substrates involving in DNA damage response such as p53 and checkpoint kinases [6]. As shown in Fig. 7a, the phosphorylation of ATM at Ser1981 was increased in A549 cells treated with C1 and C2 as well as irradiation. Furthermore, treatment with caffeine significantly decreased the compounds-induced cell death (Fig. 7b). These results suggest that mesogenic compounds induce cell death through DNA damage-signaling pathway.

We next investigated whether p53 involves in the compounds-induced cell death in A549 cells. Although knockdown of p53 decreased the C1- and C2-induced p53 protein expression in A549 cells (Fig. 7c), it did not decreased those compounds-induced cleaved caspases-3 expression and cell death (Fig. 7c and d), thus suggesting that mesogenic compounds induce cell death through DNA damage-signaling pathway, but it is independent of p53.

### Cytotoxic effects of compounds in normal human fibroblast WI-38 cells

**Fig. 8:**
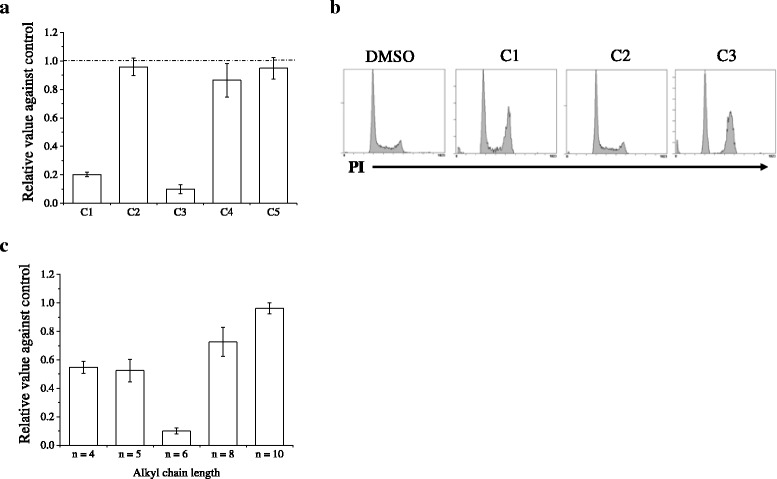
Effects of the compounds on the growth of WI-38 cells. **a** WI-38 cells cultured in the presence of test compounds at 10 μM for 3 days were harvested, and viable cells were counted using trypan blue exclusion assays. Data are presented as the mean ± SE of four independent experiments. **b** WI-38 cells cultured in the presence of test compounds (C1, C2, and C3) at 10 μM for 12 h were harvested, and then cell cycle profiles were analyzed. **c** Cytotoxic effects of compound C1 and its derivatives with varying alkyl chain lengths are shown. WI-38 cells cultured in the presence of test compounds at 10 μM for 3 days were harvested, and viable cells were counted using trypan blue exclusion assays. Data are presented as the mean ± SE of four independent experiments

In further experiments, we investigated whether our mesogenic compounds suppress the growth of WI-38 normal human fibroblast cells. As shown in Fig. 8a, C1 and C3, which carry a pyrimidine at the center of the core, dramatically suppressed WI-38 cell. In contrast, no cell growth suppression of WI-38 cells was observed with compounds C2, C4, and C5. The cell cycle analyses showed that compounds C1 and C3 induced G2/M arrest in WI-38 cells as well as NSCLC cells (Fig. 8b).

We next examined whether the alkyl chain length modulates cytotoxicity against WI-38 cells. Compound C1, which has a 6-carbon alkyl chain, caused the largest effect (Fig. 8c). Although compounds with 4- or 5-carbon alkyl chains dramatically suppressed the growth of NSCLC cells (Fig. 1f), those compounds showed only moderate cytotoxicity against WI-38 cells (approximately 40 % inhibition; Fig. 8c).

## Discussion

In this study, we investigated the cytotoxic effects of mesogenic compounds on human NSCLC cells and showed their corresponding structure–activity relationships. First, we demonstrated that 3-ring compounds carrying terminal alkyl and hydroxyl groups had superior cytotoxic effects against NSCLC cells, regardless of p53 status. Furthermore, we showed that these compounds affect cell cycle regulation lead to induction of apoptosis. In brief, compounds C1 and C3, which have a pyrimidine at the center of the core, induced caspase-mediated cell death following G2/M arrest, while compounds without a pyrimidine at the center of the core (C2, C4, and C5) induced caspase-mediated cell death following G1 arrest in a p53-independent manner.

LogP is an estimate of a compound’s overall lipophilicity, which contributes to solubility and permeability through biological membranes; there are some reports that logP is a determinant factor in the anticancer effects of some compounds [37, 38]. Supek et al. reported that the optimum logP of crown ether compounds is approximately at 5.5 for anticancer effects [38]. In the present study, logP values of our 3-ring mesogenic compounds showing cytotoxicity (C1–C5 and compounds with 4- or 5-carbon alkyl chains) ranged from 4.84 to 6.38 in NSCLC cells. Interestingly, the logP value of 3-ring compounds increased with alkyl chain lengths (the logP values of compound with 4-, 5-, 6-, 8-, and 10-carbon alkyl chains are 4.84, 5.26, 5.68, 6.52, and 7.36, respectively), and compounds with alkyl chain lengths exceeding six carbons showed lower cytotoxicity. Therefore, the lower cytotoxity of those compounds might be explained by their high lipophilicity. In contrast, compound with 4-carbon alkyl chains showed a strong cytotoxicty at 10 μM (Fig. 1f), although its logP value (4.84) was lower than the other cytotoxic compounds. However, the IC_50_ value of this compound in our previous work was approximately 5.5 μM [22], which was higher than that of C1 with 6-carbon alkyl chains (1.9 μM). These results suggest that the relatively low lipophilicity also results in low cytoxicity.

We demonstrated that C1 can induce mitotic arrest followed by cell death, thus suggesting that C1 has potential to function as antimitotic agent. Some chemotherapeutic agents targeting mitosis are clinically used or currently developed for cancer treatment [39–41]. For example, vinca alkaloids (vincrstine, vinblastine, and vindesine) or taxanes (paclitaxel and docetaxel) inhibit mitosis by disrupting microtubule dynamics [42]. In addition, the inhibition of mitotic-specific kinases (e.g., polo-like kinase 1 and aurora kinases) involving in spindle formation and mitotic checkpoint results in atimitotic effect [43]. Therefore, it is possible that C1 affects the microtubules or the mitosic-specific kinases, although we need to investigate it in future analyses.

The present study showed that 3-ring compounds carrying terminal alkyl and hydroxyl groups induced caspase-mediated cell death independent of p53. We also showed that Fas mediates the cell death induced by compounds C1 and C2. Ferreira et al. reported that anticancer drugs such as cisplatin, gemcitabine, topotecan, and paclitaxel, which are typically used in lung cancer treatment, induce apoptosis without using the Fas/Fas ligand signaling pathway [44]. Therefore, it is likely that there will be synergistic effects between our mesogenic compounds and these anticancer drugs.

Compounds C1 and C2 increased the phosphorylation of ATM, and the cell death induced by those compounds was decreased by ATM/ATR inhibitor caffeine (Fig. 7a and b). These results suggest that 3-ring compounds carrying terminal alkoxyl and hydroxyl groups may possess structurally important features for DNA damage-signaling pathway-mediated cell death that are independent of the central pyrimidine moiety. Although it is known that p53 is an important target of ATM and it plays key role in DNA damage-induced cell death [45–47], compounds C1 and C2 induced cell death in a p53-independent manner. There are several strategies that cells seem to employ to trigger p53-independent DNA damage-induced apoptosis [46, 47]. For example, a p53 homolog p73 is implicated in it. Upon DNA damage, ATM/ATR increases the levels of p73 protein through the activation of transcriptional factor E2F1 [48]. As well as p53, p73 can induce apoptosis through death receptor pathway and mitochondrial apoptotic pathway [49, 50]. Furthermore, homeodomain-interacting protein kinase 2, a serine/threonine protein kinase, also can trigger p53-indepdent DNA damage-induced apoptosis [47, 51]. Therefore, it will be necessary to determine the involvement of those proteins in the compounds-induced cell death in future study.

Side effects are considerable for most anticancer drugs. Although C1 has potential to have a broad cytotoxic effect against NSCLC cells, it also showed strong cytotoxicity against WI-38 cells. Specifically, treatment with C1 and C3 caused G2/M arrest in WI-38 cells (Fig. 8b), potentially reflecting similar cell death mechanisms following G2/M arrest as those seen in NSCLC cells. However, derivatives of C1 with 4- or 5-carbon alkyl chain lengths showed more moderate cytotoxic effects against WI-38 cells while retaining dramatic cytotoxicity against NSCLC cells, suggesting that the modulation of alkyl chain length might improve the cancer-specific cytotoxicity of these mesogenic compounds. This may be due to the differences in cell membrane compositions and cell membrane permeability between cancer and normal cells [52, 53]. On the other hand, compounds C2, C4, and C5 showed no significant growth-suppressive effects against the normal human fibroblast WI-38, which is promising for cancer-specific agents. The response to DNA damage varies depending on the cell type, and it is mentioned that fibroblasts are relatively resistant to DNA damage-induced apoptosis [45, 54]. That is why cytotoxic effect by C2 through DNA damage-signaling pathway might not be effective in human fibroblast WI-38 cells. To further clarify cancer-specific cytotoxic effects of these compounds, the cytotoxicity against other cell types such as hematopoietic cells should be examined in future study.

## Conclusions

In conclusion, this study demonstrated that not only the 3-ring structure with terminal alkyl and hydroxyl groups but also the alkyl chain lengths are structurally important for the cytotoxic effects of mesogenic compounds against human NSCLC cells. In addition, we showed that cell cycle arrest depended on the position of the pyrimidine, and that the cytotoxic effects in human lung-derived normal fibroblasts varied depending on not only the position of pyrimidine but also the terminal alkyl chain length. Although we need to verify whether the cytotoxic effects of these mesogenic compounds obtained in vitro study are effective in vivo, we believe that these structure–activity relationships will be helpful in the development of more effective and cancer-specific agents.

## Abbreviations

ATM, ataxia telangiectasia mutated; ATR, ataxia telangiectasia and Rad-3 related; CD, cluster of differentiation; DMSO, dimethyl sulfoxide; FBS, fetal bovine serum; FITC, fluorescein isothiocyanate; IC_50_, 50 % inhibitory concentration; LC, liquid crystal; NSCLC, non-small cell lung cancer; PBS(−), Ca^(2+)^- and Mg^(2+)^- free phosphate-buffered saline; PI, propidium iodide; Z-VAD-fmk, Z-Val-Ala-Asp (OMe)-CH_2_F

## Additional files

Additional file 1: Figure S1. Dose response effects of test compounds on the growth of A549 cells. A549 cells cultured in the presence of compounds C1 and C2 at 0.75–12 μM for 3 days were harvested, and viable cells were counted using trypan blue exclusion assays. Data are presented as the mean ± SE of 3 independent experiments. (DOCX 60 kb) Additional file 2: Figure S2. Effects of test compounds on cell cycle progression. Non-small cell lung cancer cells cultured in the presence of test compounds (C3–C5) at 10 μM for 24–48 h were harvested, and then cell cycle profiles were analyzed. Representative histograms are shown. (DOCX 195 kb) Additional file 3: Figure S3. Nuclear morphology of A549 cells treated with C1. A549 cells (4.0 × 10^4^ cells) were grown on chamber slides II (IWAKI) overnight to allow adherence to the slides. After treatment with compound C1 for 12 h, the cells were fixed with 4 % formaldehyde for 30 min at room temperature. Fixed cells were washed with PBS(−), permeabilized in 0.5 % TritonX-100 for 5 min at 4 °C, and washed with PBS(−). The slides were stained and mounted with VECTASHIELD® Mounting Medium with DAPI (Vector Laboratories, Inc., Burlingame, CA, USA). Photographs of the cells were taken with an Olympus IX71 (Tokyo, Japan) and DP2-BSW software (Olympus). Representative results are shown. Arrow indicates mitotic cells. (DOCX 728 kb)
