# Supplementary material for: p53-independent structure-activity relationships of 3-ring mesogenic compounds’ activity as cytotoxic effects against human non-small cell lung cancer lines
Source: BMC Cancer. 2016 Jul 25;16:521. doi: 10.1186/s12885-016-2585-6 (PMC4960859; doi:10.1186/s12885-016-2585-6)
Supplement: Additional file 1: Figure S1. — Dose response effects of test compounds on the growth of A549 cells. A549 cells cultured in the presence of compounds C1 and C2 at 0.75–12 μM for 3 days were harvested, and viable cells were counted using trypan blue exclusion assays. Data are presented as the mean ± SE of 3 independent experiments. (DOCX 60 kb) [file 12885_2016_2585_MOESM1_ESM.docx]

Additional File 1: Supplemental Figure S1

**Supplemental Figure S1** Dose response effects of test compounds on the growth of A549 cells

A549 cells cultured in the presence of compounds C1 and C2 at 0.75–12 µM for 3 days were harvested, and viable cells were counted using trypan blue exclusion assays. Data are presented as the mean ± SE of 3 independent experiments.
