# Supplementary material for: p53-independent structure-activity relationships of 3-ring mesogenic compounds’ activity as cytotoxic effects against human non-small cell lung cancer lines
Source: BMC Cancer. 2016 Jul 25;16:521. doi: 10.1186/s12885-016-2585-6 (PMC4960859; doi:10.1186/s12885-016-2585-6)
Supplement: Additional file 2: Figure S2. — Effects of test compounds on cell cycle progression. Non-small cell lung cancer cells cultured in the presence of test compounds (C3–C5) at 10 μM for 24–48 h were harvested, and then cell cycle profiles were analyzed. Representative histograms are shown. (DOCX 195 kb) [file 12885_2016_2585_MOESM2_ESM.docx]

Additional File 2: Supplemental Figure S2

**Supplemental Figure S2** Effects of test compounds on cell cycle progression

Non-small cell lung cancer cells cultured in the presence of test compounds (C3–C5) at 10 µM for 24–48 h were harvested, and then cell cycle profiles were analyzed. Representative histograms are shown.
