# Supplementary material for: p53-independent structure-activity relationships of 3-ring mesogenic compounds’ activity as cytotoxic effects against human non-small cell lung cancer lines
Source: BMC Cancer. 2016 Jul 25;16:521. doi: 10.1186/s12885-016-2585-6 (PMC4960859; doi:10.1186/s12885-016-2585-6)
Supplement: Additional file 3: Figure S3. — Nuclear morphology of A549 cells treated with C1. A549 cells (4.0 × 10^4^ cells) were grown on chamber slides II (IWAKI) overnight to allow adherence to the slides. After treatment with compound C1 for 12 h, the cells were fixed with 4 % formaldehyde for 30 min at room temperature. Fixed cells were washed with PBS(−), permeabilized in 0.5 % TritonX-100 for 5 min at 4 °C, and washed with PBS(−). The slides were stained and mounted with VECTASHIELD® Mounting Medium with DAPI (Vector Laboratories, Inc., Burlingame, CA, USA). Photographs of the cells were taken with an Olympus IX71 (Tokyo, Japan) and DP2-BSW software (Olympus). Representative results are shown. Arrow indicates mitotic cells. (DOCX 728 kb) [file 12885_2016_2585_MOESM3_ESM.docx]

Additional File 3: Supplemental Figure S3

**Supplemental Figure S3** Nuclear morphology of A549 cells treated with C1

A549 cells (4.0 × 10^4^ cells) were grown on chamber slides II (IWAKI) overnight to allow adherence to the slides. After treatment with compound C1 for 12 h, the cells were fixed with 4% formaldehyde for 30 min at room temperature. Fixed cells were washed with PBS(−), permeabilized in 0.5% TritonX-100 for 5 min at 4°C, and washed with PBS(−). The slides were stained and mounted with VECTASHIELD^®^ Mounting Medium with DAPI (Vector Laboratories, Inc., Burlingame, CA, USA). Photographs of the cells were taken with an Olympus IX71 (Tokyo, Japan) and DP2-BSW software (Olympus). Representative results are shown. Arrow indicates mitotic cells.
